# Proteomic analysis of *Rana sylvatica* reveals differentially expressed proteins in liver in response to anoxia, dehydration or freezing stress

**DOI:** 10.1038/s41598-024-65417-2

**Published:** 2024-07-04

**Authors:** Yingxi Li, Zoran Minic, Nico Hüttmann, Abdullah Khraibah, Kenneth B. Storey, Maxim V. Berezovski

**Affiliations:** 1 Department of Chemistry and Biomolecular Sciences, University of Ottawa, Ottawa, ON K1N 6N5 Canada; 2 John L. Holmes Mass Spectrometry Facility, Faculty of Science, University of Ottawa, Ottawa, ON K1N 6N5 Canada; 3 Department of Biology, Carleton University, 1125 Colonel By Drive, Ottawa, ON K1S 5B6 Canada

**Keywords:** Enzymes, Proteomics, Mass spectrometry

## Abstract

Ectothermic animals that live in seasonally cold regions must adapt to seasonal variation and specific environmental conditions. During the winter, some amphibians hibernate on land and encounter limited environmental water, deficient oxygen, and extremely low temperatures that can cause the whole body freezing. These stresses trigger physiological and biochemical adaptations in amphibians that allow them to survive. *Rana sylvatica*, commonly known as the wood frog, shows excellent freeze tolerance. They can slow their metabolic activity to a near halt and endure freezing of 65–70% of their total body water as extracellular ice during hibernation, returning to normal when the temperatures rise again. To investigate the molecular adaptations of freeze-tolerant wood frogs, a comprehensive proteomic analysis was performed on frog liver tissue after anoxia, dehydration, or freezing exposures using a label-free LC–MS/MS proteomic approach. Quantitative proteomic analysis revealed that 87, 118, and 86 proteins were significantly upregulated in dehydrated, anoxic, and frozen groups, suggesting potential protective functions. The presence of three upregulated enzymes, glutathione S-transferase (GST), aldolase (ALDOA), and sorbitol dehydrogenase (SORD), was also validated. For all enzymes, the specific enzymatic activity was significantly higher in the livers of frozen and anoxic groups than in the controls. This study reveals that GST, ALDOA, and SORD might participate in the freeze tolerance mechanism by contributing to regulating cellular detoxification and energy metabolism.

## Introduction

Many animals show extraordinary resilience in the face of harsh environmental conditions, showcasing their remarkable ability to adapt and survive under diverse conditions^1^. Through the process of natural selection, species have developed an amazing array of adaptative strategies that enable them to thrive in challenging habitats, ranging from the Arctic tundra to scorching deserts. Under extreme cold conditions, these can include adaptations such as thick fur or insulating blubber to conserve body heat among mammals or, oppositely, the use of heat-dissipating mechanisms in order to regulate body temperatures within a narrow range^2–5^. Strong metabolic rate depression is also widespread to minimize rates of energy production and consumption in order to cope with limited food resources or extreme temperatures^6,7^. Behavioral adaptations, such as migration, hibernation, or burrowing, also aid various species in avoiding unfavorable conditions^8–10^. Furthermore, the ability to enter states of dormancy or suspended animation allows many species to survive through periods of environmental extremes (e.g. heat, cold, drought, etc.)^11,12^.

The present study focuses on the wood frog, *Rana sylvatica* (also known as *Lithobates sylvaticus*), that stands out as one of Canada's most intriguing amphibians due to its amazing capacity for freeze tolerance. Wood frogs are widely distributed across diverse habitats, from the southern Appalachian Mountains to the boreal forests as far north as the Arctic Circle^13^. These adaptable frogs have mastered the art of survival under Canada's varying climates. One of the most astonishing aspects of their resilience is their remarkable ability to tolerate the freezing of up to 65–70% of their total body water as extracellular ice^14^. When they freeze, their cells face challenges of severe dehydration caused by water loss into extracellular ice masses and extended periods of oxygen deprivation due to freezing of blood plasma, that interrupts oxygen supply to organs^15^. Consequently, these frogs have developed tolerances for both cell/tissue dehydration and anoxia as adaptations to cope with harsh winter conditions. Most essential physiological activities, such as respiration, heartbeat, muscle function, and brain activity, are strongly slowed or entirely halted during freezing^16^.

In addition to physiological adaptations, wood frogs exhibit multiple biochemical adaptations for winter survival. They achieve their remarkable freeze tolerance via two main strategies: (1) the production of high concentrations of cryoprotectants and (2) the regulation of ice recrystallization^15^. Under the subzero temperatures of winter, extracellular water freezes causing a high proportion of intracellular water to be drawn out of cells to comply with osmosis. As a result, cells lose a high percentage of their water, but to prevent cells and organs from being damaged by dehydration, wood frogs and other freeze-tolerant species produce high concentrations of cryoprotectants that are packed into their cells (e.g., glucose, glycerol, sorbitol, or urea) that act as natural antifreeze. In addition, cells and tissues can be physically damaged by ice crystals penetrating cell membranes. To protect their intracellular environment, wood frogs can also produce ice-binding proteins to restrict the formation and maintenance of ice crystals to extracellular and extra-organ compartments only^15^.

In addition to the production of high concentrations of cryoprotectants and ice-binding proteins, metabolic modifications also contribute to freeze tolerance. Wood frogs generally adapt their metabolism for fuel and energy preservation during the winter^17^. During this period, the overall metabolic activities of these ectotherms decrease strongly as temperature falls, leading to a substantial reduction in energy expenditure^18^. This slowdown in metabolism helps conserve fuel reserves, allowing frogs to survive the winter without food intake. In addition, some metabolic processes, such as detoxification, can be preserved as part of the stress response^19^.

With their extraordinary freeze tolerance, wood frogs can survive being frozen solid for weeks or months. Then, with the arrival of spring, they thaw out and resume their normal activities with little or no tissue damage^16^. This unique survival strategy has captured the attention of many scientists and researchers, leading to extensive studies aimed at unraveling the biological mechanisms behind this incredible feat. However, unlike commonly used model organisms such as humans and rats with well-annotated genomes, non-model organisms, such as wood frogs, lack extensive genetic information, making traditional genetic studies challenging. Mass spectrometry-based proteomics has emerged as a powerful tool in studying non-model organisms, expanding our understanding of their biology and adaptation to diverse environments^20^. By analyzing the proteome, researchers can gain insights into protein expression patterns, post-translational modifications, protein–protein interactions, and signaling pathways, providing valuable information about the organism's responses and adaptations to environmental stress.

In the present study, we undertook protein profiling of wood frog liver using MS-based proteomics approaches to understand further metabolic processes that contribute to freeze tolerance. Liver tissue was harvested from wood frogs after exposure to anoxia, dehydration, or freezing stress conditions. Tissue samples were analyzed by nano-liquid chromatography-tandem mass spectrometry (nLC-MS/MS), and protein profiles were compared with those of 5 °C control frogs. The results revealed multiple differentially expressed proteins. Three significantly upregulated proteins were chosen for validation and deeper analysis.

## Materials and methods

### Ethical statement

The study is reported in accordance with ARRIVE guidelines. Ethical procedures were strictly adhered to, with all animal protocols having received prior approval from the Carleton University Animal Care Committee (Protocol #13683), and following the guidelines set by the Canadian Council on Animal Care. The collection of wood frogs was authorized under Wildlife Scientific Collector's Authorization #1085726 granted by the Ministry of Natural Resources of Ontario^21^. Groups of 18 frogs were prepared for each treatment group. Frogs in all groups were humanely euthanized using approved methods, followed by prompt dissection of the liver first (samples of other tissues then followed). Liver samples were immediately frozen in liquid nitrogen and then preserved at − 80 °C until required for analysis.

### Biological sample preparation

Adult wood frogs (~ 5–7 g) were collected from early spring breeding ponds in the Ottawa, Ontario region and transferred to the lab in coolers containing snow or crushed ice. In the lab, all frogs were given a bath in cold water containing tetracycline and were then housed in opaque plastic boxes (12 × 10 × 3 inches) lined with damp sphagnum moss and transferred into an incubator at 5 °C for a 2-week acclimation period. Frogs were checked daily. The control group was sampled randomly from this condition after the acclimation period^21–23^.

Dehydration exposure was conducted as previously described^23^. Briefly, acclimated frogs were individually weighed, and then 5–6 frogs of diverse initial weights were placed in each of several tall, opaque, and dry plastic buckets in a 5 °C cold incubator. Under these conditions, frogs slowly lost body water via evaporation across the skin. Frog weights were initially recorded twice a day and more frequently as the target water loss was approached. Calculation of the amount of body water lost was performed using the following equation:

$$\% \text{ Dehydration }= \frac{({M}_{i} - {M}_{d})}{({M}_{i} - {BWC}_{i})} \times 100 \%$$

where M_i_ is the initial body mass of the frog, M_d_ is the mass after experimental dehydration, and BWC_i_ is the initial body water content of frogs before dehydration treatment. The BWC_i_ was previously determined to be 80.8 ± 1.2% g of H_2_O per g body mass^23^. Water loss was discontinued when frogs reached approximately 40% dehydration.

Anoxia exposure was conducted following a previously published protocol^22^. Briefly, acclimated frogs were placed in cold containers (held in crushed ice) that had been pre-flushed with nitrogen gas via ports in the lid for 30 min. Frogs were then quickly added (typically 5 per container), and then the container was flushed again with nitrogen gas for 30 min. Ports were sealed, and containers were transferred back to the 5 °C incubator for 24 h. After 24-h exposure under a nitrogen gas atmosphere, containers were returned to a crushed ice container, the nitrogen gas line was reconnected, and frogs were rapidly sampled.

For freezing exposure, small groups of frogs were placed in plastic containers lined on the bottom with damp paper towels. Animals were held at − 4 °C in an incubator for 45 min to trigger ice nucleation in their bodies due to contact with ice on the paper towel. Temperature was then raised to − 2.5 °C, and frogs were held for 24 h at − 2.5 °C followed by quick sampling^21^.

### Tissue homogenization

Frozen liver tissue samples were each suspended in 0.5 mL of ice-cold lysis buffer and homogenized for 3 min. Lysis buffer consisted of 1.5 M urea, 5% (v/v) glycerol, 1 mM DTT, 1:200 (v/v) protease inhibitor cocktail (Thermo Scientific, #78430), and 25 mM HEPES at pH 8.0. The suspension was centrifuged at 12000*g* for 10 min at 4 °C, and soluble protein supernatants were separated from insoluble debris. Samples of 50 μL of the resulting supernatant were used for protein quantification, and 150 μL of the supernatant was used for proteomic sample preparation.

### Protein quantification by Bradford assay

The protein concentration and quantity of the resulting supernatant were determined by the Bradford assay^24^ using a protein assay kit (#23200, Thermo Fisher Scientific) following the manufacturer’s protocol. The assay absorbance was measured with a spectrophotometer (Novaspec III, Biochrom) and semi-microvolume disposable polystyrene cuvettes (#2239955, Bio-Rad) at 595 nm.

### Protein reduction, alkylation and enzymatic digestion

Aliquots of 50 µg of protein were further processed with a modified filter-aided sample preparation (FASP) protocol for proteomic analysis. The resulting supernatant samples containing 50 µg of protein were diluted to a total volume of 200 µL with a denaturation buffer (8 M urea, 25 mM HEPES at pH = 8.0). The samples were vortexed briefly and transferred to a 10 kDa MWCO filter (MRCPRT010, Millipore). The sample volume was reduced to about 20 µL by centrifugation for 15 min at 14,000*g*, and proteins were reduced by the addition of 4 mM Tris(2-carboxyethyl) phosphine (TCEP) in 100 µL denaturation buffer. The samples were then incubated at 25 °C for 30 min, followed by a 15-min centrifugation at 14,000*g*. Proteins were then alkylated with 20 mM of iodoacetamide (IAA) in 100 µL denaturation buffer. The samples were incubated at 25 °C for 40 min, followed by a 15-min centrifugation at 14,000*g*. Next, 100 µL of digestion buffer (0.6% v/v glycerol, 25 mM HEPES, pH = 8.0) was added into the filter, followed by a 15-min centrifugation at 14,000*g*. After buffer exchange, the filter was transferred to a clean collection tube. Proteolytic digestion was performed by the addition of MS-grade trypsin/Lys-C mix (#V5072, Promega), 1:150 enzyme to protein ratio, and incubated in the dark with shaking at 600 rpm at 37 °C for 12 h. Peptides were eluted as filtrate by centrifugation at 14,000*g* for 15 min, and 2% (v/v) of formic acid was added to the collected filtrates to stop digestion. The collected filtrate containing about 50 µg of protein was desalted on disposable TopTip C-18 micro-spin columns (#TT2C18.96, Glygen, Ellicott City, MD, USA) and dried by vacuum centrifugation (Savant SPD111V SpeedVac Concentrator, Thermo Scientific).

### Nano-LC–MS/MS

Protein samples were analyzed by an Orbitrap Fusion mass spectrometer (Thermo Fisher Scientific) coupled to an UltiMate 3000 nanoRSLC (Dionex, Thermo Fisher Scientific). One microliter of peptides (equivalent to 2.0 μg of protein) was loaded onto an in-house packed column (Polymicro Technology), 15 cm × 70 μm ID, Luna C18(2), 3 μm, 100 Å (Phenomenex) employing a water/acetonitrile/0.1% formic acid gradient. Peptides were separated for 105 min at a flow rate of 0.30 μL/min and the following steps: 0–7 min, 2–2% ACN; 7–77 min, 2–38% ACN; 77–82 min, 38–98% ACN; 82–92 min, 98–98% ACN; 92–95 min, 98–2% ACN; 95–105 min, 2–2% ACN. Eluted peptides were directly sprayed into a mass spectrometer using positive electrospray ionization (ESI) at an ion source temperature of 250 °C and an ion spray voltage of 2.1 kV. The Orbitrap Fusion mass spectrometer was run in data-dependent MS/MS acquisition following a full MS survey scan in top-speed mode. Full-scan MS spectra (m/z 350–2000) were acquired at a resolution of 60,000. Precursor ions were filtered according to monoisotopic precursor selection, charge state (+ 2 to + 7), and dynamic exclusion (30 s with a ± 10 ppm window). The automatic gain control settings were 5 × 10^5^ for full FTMS scans and 1 × 10^4^ for MS/MS scans. Fragmentation was performed with collision-induced dissociation (CID) in the linear ion trap. Precursors were isolated using a 2 m/z isolation window and fragmented with a normalized collision energy of 35%.

### MS spectra processing

Mass spectrometry raw data files of 72 samples were analyzed using MaxQuant, version 1.6.4.0^25^. Peptides were searched against the *Lithobates* genus (NCBI taxonomy ID: 192752) FASTA file containing 30,829 reviewed and unreviewed entries (09.04.2021) and a default contaminants database using the Andromeda search engine, integrated into MaxQuant^26^. Default parameters were used unless stated otherwise. Methionine oxidation and N-terminal acetylation were set as variable modifications, while cysteine carbamidomethylation was set as a fixed modification. Trypsin and LysC proteases were chosen as the digestion enzyme-generating peptides of at least 7 amino acids with a maximum of 2 missed cleavages. A false discovery rate (FDR) was set to 0.01 for peptides and proteins, which were determined using a reverse sequence decoy database. A contaminant database provided by the Andromeda search engine was used. Peptides were identified with an initial precursor mass deviation and a fragment deviation of up to 10 ppm, and 0.5 Da, respectively. To increase the peptide identification rate, the ‘Match between runs’ algorithm in MaxQuant was performed between all samples^27^. Proteins and peptides matching to the reverse database or identified as contaminant were filtered out. A minimum ratio count of 2 was required for label-free quantification. Label-free quantification (LFQ) values were obtained through MaxQuant quantitative label-free analysis.

### Data filtering

The MaxQuant output tables *proteinGroups.txt* were loaded in R and analyzed using an in-house pOmics R package (github.com/nicohuttmann/pOmics). Potential contaminants and reverse proteins annotated by MaxQuant were excluded from the analysis. Principle component analysis (PCA) was generated using the prcomp R function based on the imputed label-free quantification (LFQ) intensity values. Proteins with detected LFQ intensities in 50% of the samples were used for imputation, and imputation of missing LFQ intensities was based on a down-shifted Gaussian distribution of log-transformed protein LFQ intensities [shift = 1.8 standard deviations (sd), width = 0.3 sd], stimulating low abundance profiles^28^. The log_2_ (fold-change) values and p-values by unpaired t-test for proteins were computed using the imputed LFQ intensities. The GO enrichment analysis was made by enriching the detected proteins against the *Lithobates catesbeianus* (NCBI taxonomy ID: 8400) database.

### Glutathione S-transferase (GST) activity assay

Glutathione S-transferase activity was measured using a glutathione S-transferase activity assay kit (ab65326, Abcam, Toronto, ON, Canada) according to the manufacturer’s procedures. Frozen liver tissue samples from four wood frog groups were resuspended in 0.5 mL of ice-cold 1 × phosphate buffer saline and homogenized for 3 min. The suspension was centrifuged at 12,000*g* for 10 min at 4 °C, and supernatants were collected and used for measurement of enzymatic activities. Assays were performed at 22 °C, and absorbances were measured at 450 nm in a kinetic mode according to the manufacturer’s instructions. Enzyme activities were calculated from the assay time of 40 min using the extinction coefficient factor provided by the standard operating procedure.

### Aldolase (ALDOA) activity assay

Aldolase activity was measured using an aldolase activity assay kit (ab196994, Abcam, Toronto, ON, Canada) according to the manufacturer’s procedures. Supernatant samples collected from liver tissue homogenates prepared initially for GST activity measurement were also used for the aldolase activity assay. Assays were performed at 25 °C, and absorbances were measured at 340 nm in a kinetic mode according to the manufacturer’s instructions. The activity of enzymes was calculated from the assay time of 40 min via the calibration curve of the NADH standard at known concentrations (Fig. S1A).

### Sorbitol dehydrogenase (SORD) activity assay

Sorbitol dehydrogenase activity was measured using a sorbitol dehydrogenase activity assay kit (ab252902, Abcam, Toronto, ON, Canada) according to the manufacturer’s procedures. Supernatants collected from liver tissue homogenates for GST activity measurement were used for the sorbitol dehydrogenase activity assay. Assays were performed at 25 °C, and absorbances were measured at 450 nm in a kinetic mode according to the manufacturer’s instructions. Enzyme activities were calculated from the assay time of 40 min via the calibration curve of the NADH standard at known concentrations (Fig. S1B).

## Results

### Liver protein profiling upon external stressors

**Figure 1:**
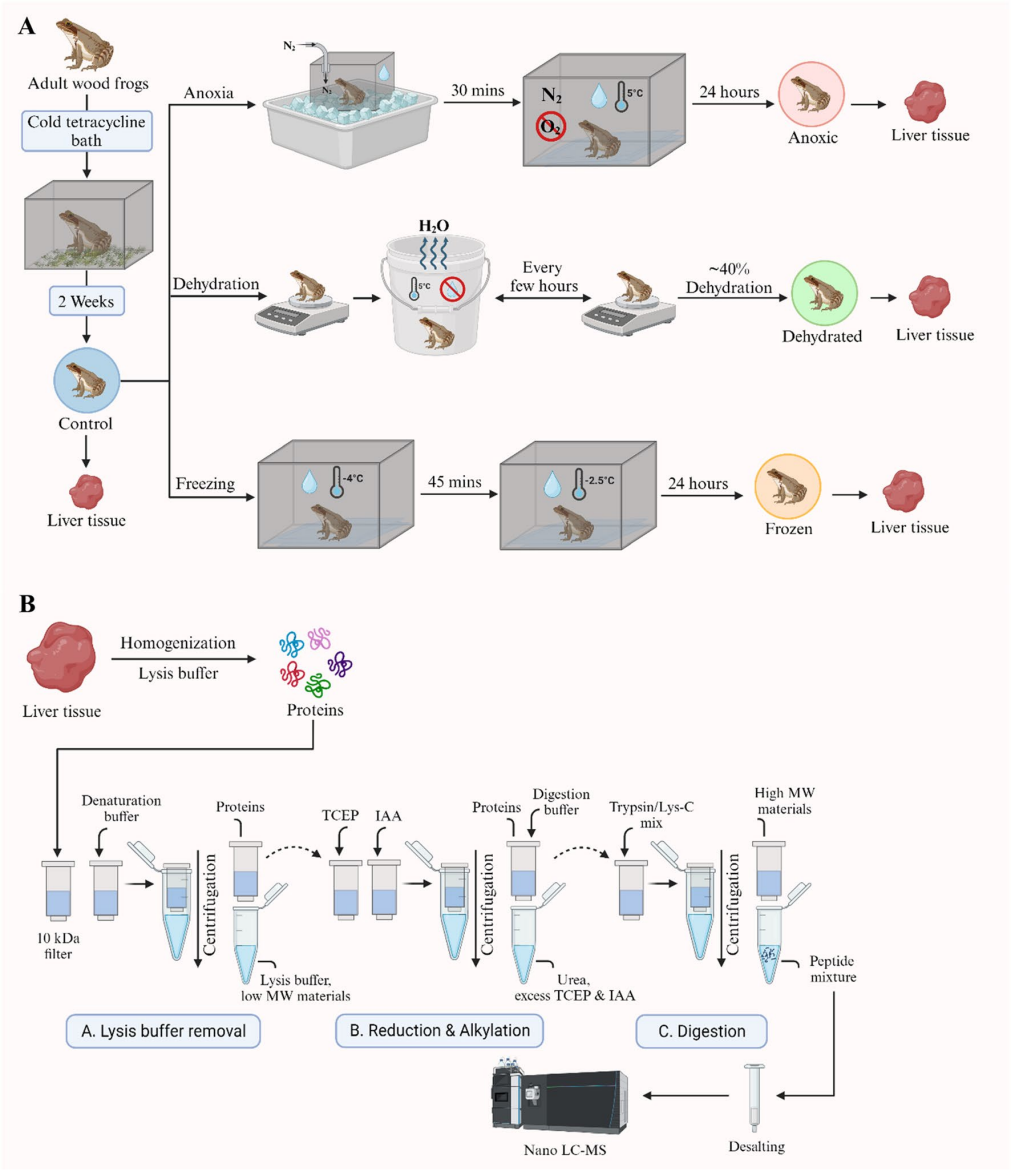
Workflow of proteomic sample preparation. (**A**) Frogs were treated under different conditions: control, anoxic, dehydrated, and frozen exposures. Liver tissues were sampled after the treatment and used for proteomic analysis. (**B**) Proteomic sample preparation was performed using the FASP method. Extracted proteins from frog liver tissues were reduced, alkylated, and digested into peptides and then analyzed with nLC-MS/MS.

Wood frog liver tissue samples containing approximately 50 μg of proteins were prepared from control, anoxic, dehydrated, and frozen groups. Eighteen biological replicates were obtained per group and examined independently. Proteins were digested using a FASP method that efficiently removes impurities from metabolites and consequently can increase the number of proteins identified^29^. A schematic overview of our experimental strategy is presented in Fig. 1.

**Figure 2:**
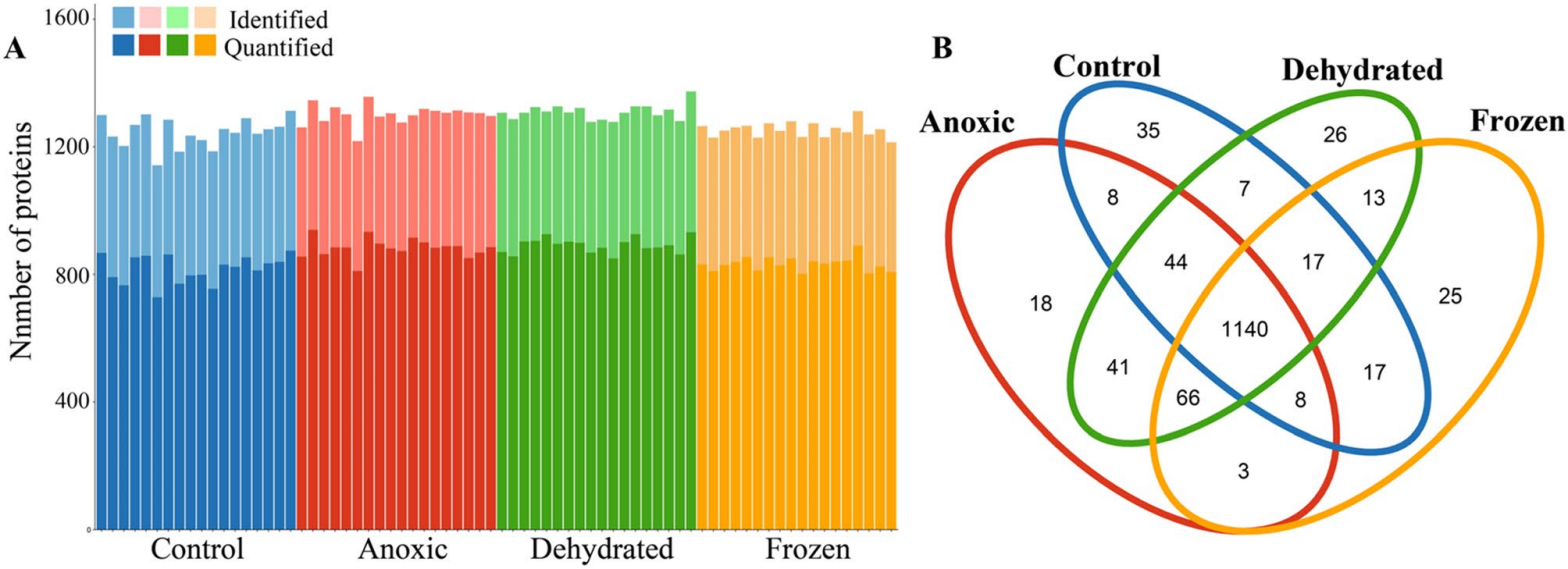
Overview of the identified and quantified proteins. (**A**) The total number of identified and quantified proteins from control, anoxic, dehydrated, and frozen groups. (**B**) A Venn diagram showing the number of overlapping proteins from the four groups.

Peptides obtained after digestion were separated and analyzed using nano-liquid chromatography-tandem mass spectrometry (nLC-MS/MS). Rigorous bioinformatics analysis and protein identification were applied to the proteomic mass spectrometry data. Proteins were identified and quantified using MaxQuant search engine using the *Lithobates* genus (NCBI taxonomy ID: 192752) database. Proteins were analyzed with strict criteria to ensure high confidence with a false discovery rate of < 1%. After the removal of reverse sequences and potential contaminants, a list of all identified proteins along with relevant parameters for protein identification is presented in Table S1. The total number of identified and quantified proteins for each group is also presented in Fig. 2A and Table S2. In total, 1245, 1301, 1308, and 1253 proteins were identified, and 817, 883, 891, and 833 proteins have been quantified in control, anoxic, dehydrated, and frozen groups, respectively (Fig. 2A). The Venn diagram of proteins revealed that 1140 identified proteins were found in all four groups, and less than 40 proteins were found as unique proteins for each group (Fig. 2B).

### Clustering analysis of proteins

**Figure 3:**
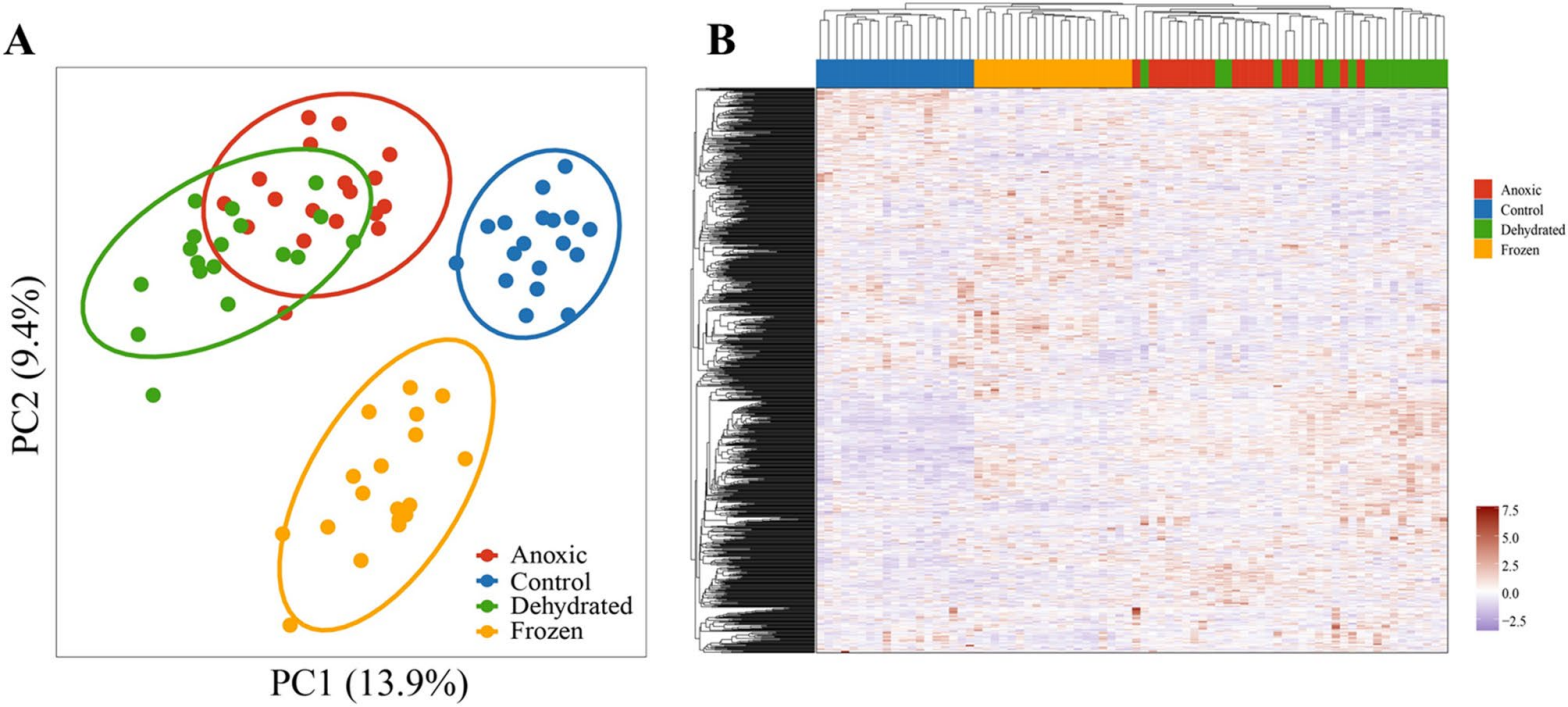
Clustering analysis of identified proteins. (**A**) Principal component analysis of samples from control, anoxic, dehydrated, and frozen groups. (**B**) Heatmap analysis of samples from the four groups.

A principal component analysis (PCA) and a hierarchical clustering analysis were performed to determine group similarity. The generation of principal component analysis (PCA) and hierarchical clustering analysis was based on imputed LFQ intensities for which proteins needed to be present in at least 50% of the samples in one group. The PCA and the hierarchical clustering analysis showed that there were clear differences between the control group and the three other groups (Fig. 3). Furthermore, the anoxic and dehydrated groups appear to have a high degree of similarity in their protein profile as their clusters were partially overlapped.

### Visualization of protein abundance

**Figure 4:**
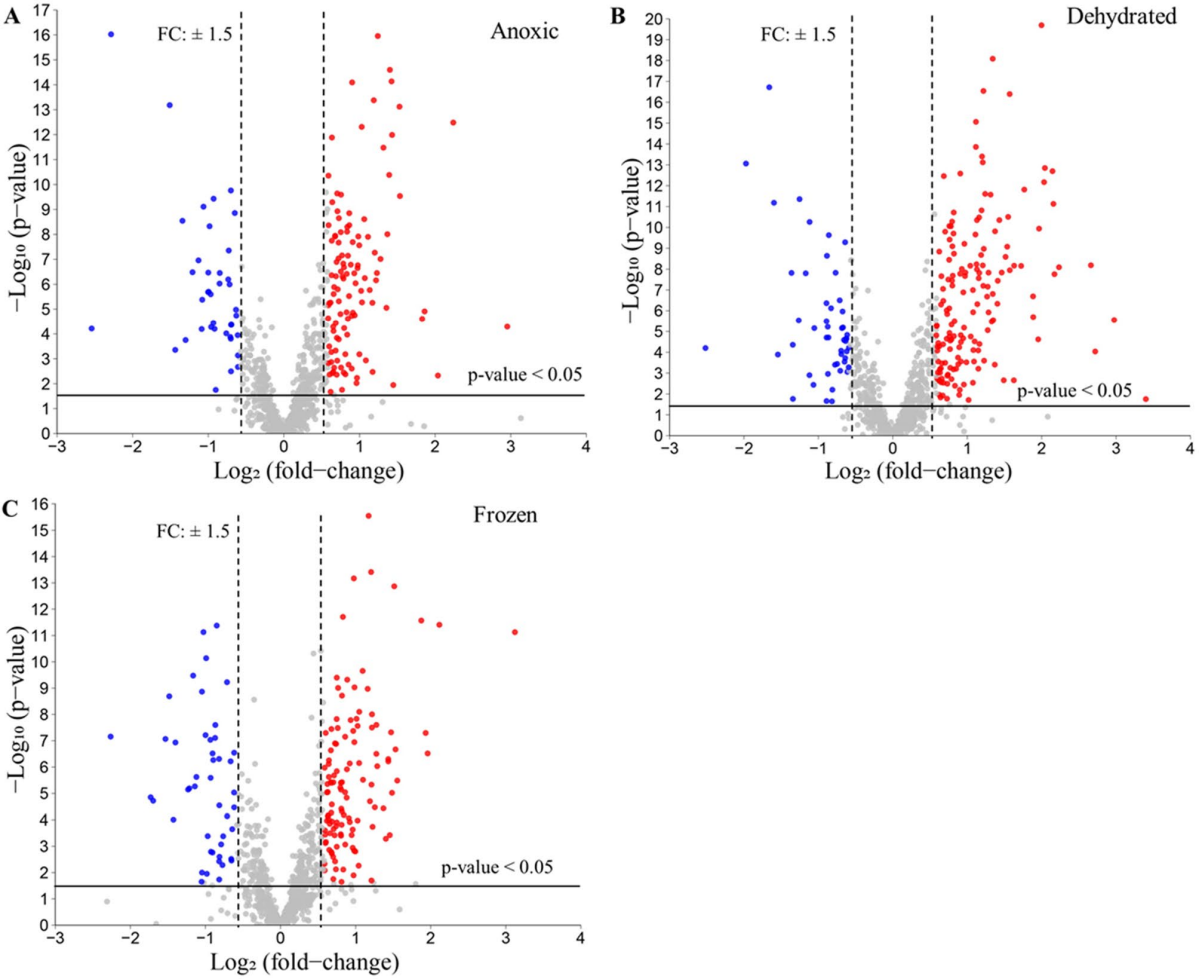
Volcano plots of quantified proteins from liver. Protein quantities between the three conditions and the control group were compared. Red dots indicate proteins that were upregulated under the stress condition; blue dots indicate proteins that were downregulated under the stress condition; grey dots show proteins that did not change significantly between control and stress conditions. The analysis threshold was p-value < 0.05 and the fold change (FC) > 1.5 or < 0.67; anything below these thresholds was considered not significant. (**A**) Volcano plot of anoxic vs. control; (**B**) Volcano plot of dehydrated vs. control; (**C**) Volcano plot of frozen vs. control.

Volcano plots were produced to visualize the differentially expressed proteins in each treatment group (Fig. 4). Fold changes (FC) calculated by LFQ protein intensity and t-tests for p-values were implemented. FC > 1.5 or < 0.67 and p-values < 0.05 were considered thresholds to identify differentially expressed proteins (DEPs). Overall, 113, 156, and 118 proteins were identified as significantly upregulated in anoxic, dehydrated, and frozen groups, respectively, as compared to the control group. The complete list of upregulated, downregulated, and non-significantly differentially expressed proteins is presented in Table S3. In this study, we focus on upregulated proteins to identify molecular markers that are associated with particular stress conditions.

Figure S2 shows Venn diagram analysis of up- and down-regulated proteins extracted from the results of Volcano plot (Fig. 4, Table S3). For the up-regulated proteins, 18, 34 and 47 proteins were unique for anoxic, dehydrated and frozen conditions, respectively. An important number of up-regulated proteins overlap between these three groups (69 proteins). For the down-regulated proteins, 17, 29 and 38 proteins were unique for anoxic, dehydrated and frozen conditions, respectively. There was not a large overlap between these three conditions, but a significant amount of overlap is observed between dehydrated and anoxic groups.

### Functional analysis of significantly upregulated proteins

**Figure 5:**
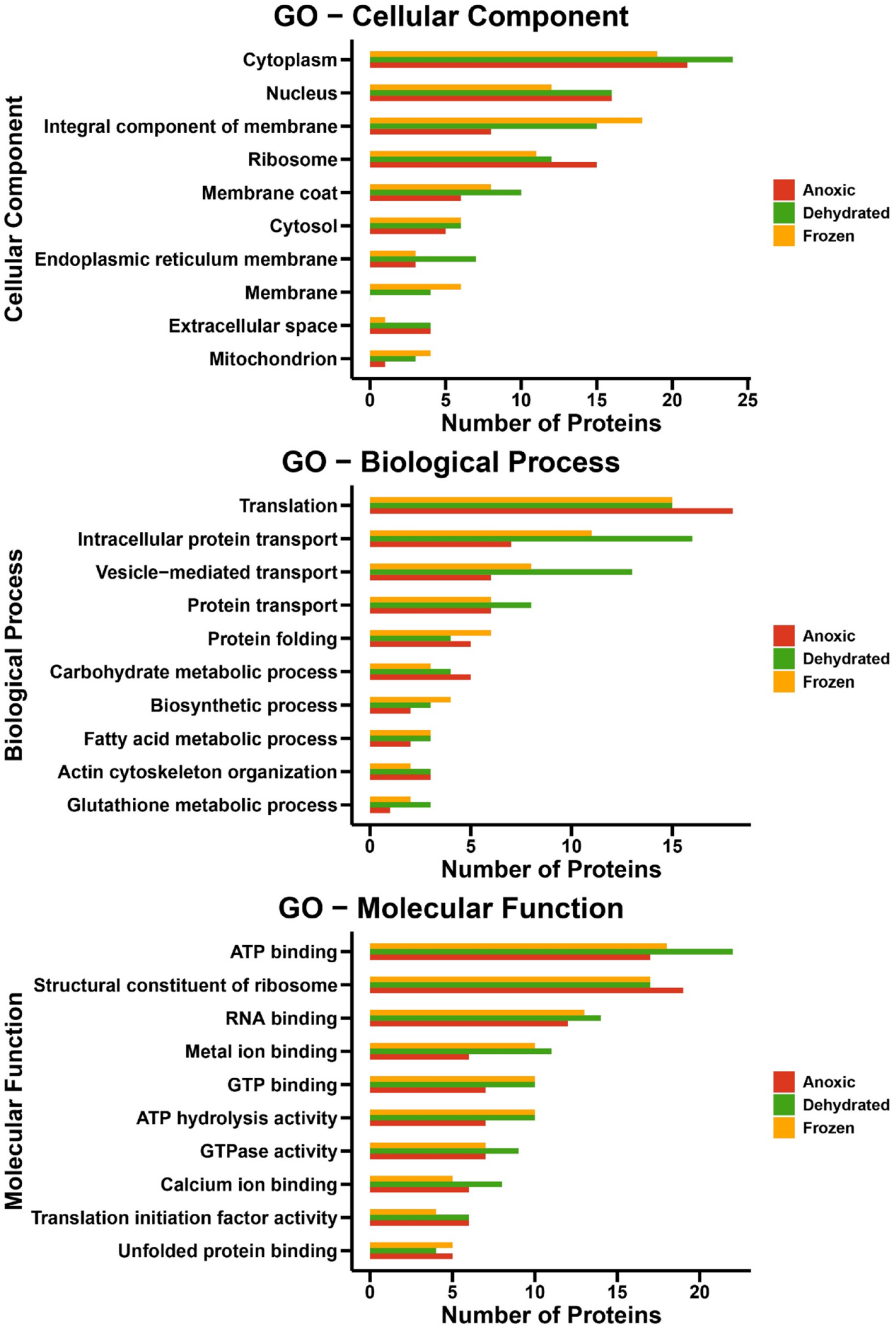
Top 10 Gene Ontology (GO) functional annotation labels for upregulated proteins in anoxic, dehydrated, and frozen groups. (**A**) Cellular Components, (**B**) Biological processes, and (**C**) Molecular functions.

Gene Ontology (GO) analysis was used to annotate the significantly upregulated proteins in anoxic, dehydrated, and frozen groups as compared to the control group (Fig. 5). The GO enrichment analysis of up-regulated proteins used cellular components, biological processes, and molecular function terms. Within the GO cellular component terms, the upregulated proteins were mainly localized to the cytoplasm, nucleus, ribosomes, and integral component of membrane categories. The GO biological process was enriched mainly in the term Translation, but several other terms related to transport were also enriched, including intracellular protein transport, vesicle-mediated transport, and protein transport. Interestingly, the dehydrated group had more enriched proteins related to protein transport as compared to the other treatment groups. Within the GO molecular function terms, the upregulated proteins were mainly annotated as structural constituents of ribosome, ATP binding, RNA binding, and ATP hydrolysis activity. The results for these three stress conditions show an overlap between the GO of cellular components, biological processes, and molecular function terms. However, the number of proteins for each condition and term differed. Enrichment analysis of GO terms for diverse stress and response conditions revealed an unexpectedly low number of proteins (Table 1).

**Table 1:** GO enrichment analysis of upregulated proteins in anoxic, dehydrated, and frozen groups in response to diverse stress and response conditions.

Upregulated enzymes	GO for stress-responsive terms	Conditions
Eosinophil peroxidase	Defense response [GO:0006952]	Anoxic, dehydrated
Epoxide hydrolase	Response to toxic substance [GO:0009636]	Dehydrated, frozen
Aconitate hydratase	Response to iron (II) ion [GO:0010040]	Frozen

Gene Ontology (GO) enrichment analysis of downregulated proteins in anoxic, dehydrated, and frozen groups as compared to the control group is presented in Fig. S3. Although this analysis was performed on a small number of proteins (39, 49 and 46 proteins in the anoxic, dehydrated, and frozen groups, respectively), it appears that there are substantial differences in the terms for GO enrichments in comparison to upregulated protein. It is interesting to note that tricarboxylic acid cycle for all three conditions were affected. Therefore, two proteins of tricarboxylic acid cycle were found to be downregulated in frozen, dehydrated and anoxic conditions. These proteins include malate dehydrogenase and oxoglutarate dehydrogenase.

### Significant differentially expressed enzymes

Since metabolic changes play a pivotal role in the adaptation of wood frogs to extreme environments^30^ and this change can be easily monitored by enzymatic assay to assess these metabolic alterations, the significantly upregulated protein list was further narrowed by selecting only enzymes. In the upregulated protein list, 16, 28, and 20 proteins are enzymes in the anoxic, dehydrated, and frozen groups, respectively. The list of significantly upregulated enzymes can be found in Table S4. Some of these enzymes have a molecular function that can be linked to the wood frog’s freeze tolerance mechanism (Table 2). In addition to the enzymes shown in Table 2, proteomic analysis identified aldolase as a unique protein present only in the anoxic group.

**Table 2:** Significantly upregulated enzymes in anoxic, dehydrated, and frozen groups compared to the control group, and their molecular functions.

Upregulated enzymes	Molecular function	Fold change		
		Anoxic	Dehydrated	Frozen
Glutathione S-transferase (GST)	Detoxification of ROS	3.6	4.5	8.7
Sorbitol dehydrogenase (SORD)	Carbohydrate metabolism	1.8	1.7	–
Epoxide hydrolase (EH)	Metabolism of xenobiotics	–	2.1	3.8
Sulfotransferases (ST)	Detoxification	3.6	3.9	–
Lactate dehydrogenase (LDH)	Anaerobic metabolism	2.3	2.5	–
Heme oxygenase (HO)	Oxidative stress response	–	–	1.9

“–” represents that enzymes were not significantly upregulated in such groups.

### Validation of specific activity of GST, ALDOA, and SORD

**Figure 6:**
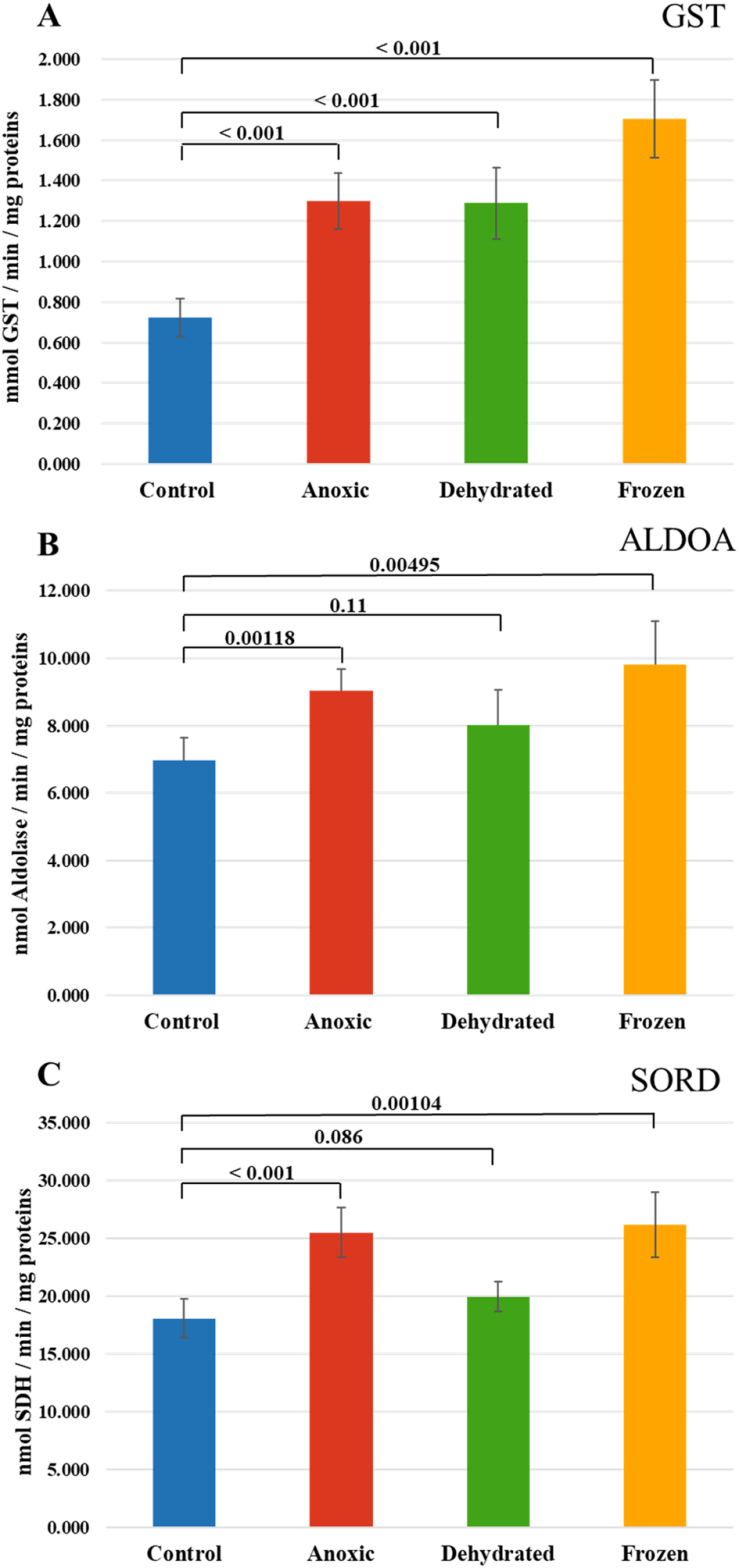
Specific enzymatic activity of (**A**) Glutathione S-transferase (GST), (**B**) Aldolase (ALDOA), and (**C**) Sorbitol dehydrogenase (SORD) from frog liver. The bar graph represents the mean value of the biological replicates, whereas error bars indicate the standard deviation of the replicates, and p-values obtained from a Student’s t-test are indicated on the top. Data were considered statistically significant if p < 0.05.

Three significantly upregulated enzymes identified by the proteomics approach were selected for further analysis: glutathione S-transferase (GST), aldolase (ALDOA), and sorbitol dehydrogenase (SORD). Enzymatic kits for other enzymes presented in Table 1 were not commercially available and consequently further analyses of these enzymes were not conducted. Enzymatic activities were tested in frog liver tissues for each enzyme using five biological replicates. The specific activities were measured in nmol per minute per milligram of proteins, and the mean specific activities of biological replicates are presented in Fig. 6. The difference in specific activity was considered statistically significant if the p-value < 0.05. The specific activity levels of all tested enzymes were significantly higher in the frozen and anoxic groups using t-test analysis as compared with the control group. Similarly, the specific activity of GST was significantly higher in the dehydrated group as compared with the control group, but ALDOA and SORD did not show significantly different specific activities among the two groups. To provide more information regarding multiple comparisons between groups, the results of one-way analysis of variance (ANOVA) are performed (Fig. S4). The results confirmed significantly different specific activities among the four groups.

## Discussion

The field of freeze tolerance among animals has been explored since the 1960s^31,32^. In its early stages, the field was limited in scope, primarily concentrating on essential data collection about freeze-tolerant organisms, including information on species variety, freeze survival parameters, ice nucleators, and identification of cryoprotectants^33^. The field has now grown significantly, and studies from different aspects have been conducted among many animal groups^15^. The wood frog, *Rana sylvatica*, is the best-researched freeze-tolerant vertebrate and serves as the primary model for investigating the molecular, biochemical, and physiological mechanisms underlying freezing survival^16^. MS-based proteomics is considered a powerful tool for investigating the molecular adaptations of organisms for survival under environmental stresses of many kinds. However, there have been limited proteomic studies conducted on wood frogs. Kiss et al*.* performed a proteomic analysis on wood frogs collected during both summer and winter, identifying 33 proteins with significant seasonal variations. Their results revealed that winter frogs exhibited elevated expression of proteins associated with cryoprotection, whereas proteins involved in cell proliferation, protein synthesis, and mitochondrial function showed reduced abundance^34^. Hawkins et al*.* employed phosphoproteome analysis to investigate the phosphorylation of metabolic enzymes in wood frogs. Their study focused on enzymes related to glucose and urea production, specifically in response to freezing, anoxia, or dehydration exposures. The study revealed distinct stress-specific variations in phosphopeptide abundance of 9 glycolytic enzymes and 3 urea cycle enzymes in the liver of wood frogs^21^.

A shotgun label-free quantitative proteomic approach was performed in our study to understand the molecular basis of freeze tolerance in wood frogs. The results of this analysis showed unique proteome patterns and many differentially expressed proteins under different stress conditions, including anoxia, dehydration, and frozen groups. It is important to note that alteration in protein abundance is not a necessary consequence in gene expression and can be regulated at multiple levels including post-transcriptional, translational and protein degradation regulation. As ectotherms, the metabolic activities of wood frogs dramatically slow in the winter^30^, although a few activities rise as part of the cold stress response. Previous studies showed that winter wood frogs (when triggered by ice formation on their skin) immediately activate liver glycogenolysis to produce copious amounts of glucose that is then exported as a cryoprotectant and rapidly taken up by all tissues^35,36^. These high sugar concentrations mitigate cell/tissue damage from excessive water loss into extracellular ice masses. Antioxidant defenses and pro-survival pathways are also enhanced^37,38^. Since ATP provides the energy needed for many essential processes in cells and organisms^30,39, 40^, it is not surprising that the GO molecular function term “ATP hydrolysis activity” was significantly enriched among upregulated proteins. Interestingly, the dehydration group was mostly enriched in the GO biological process terms “intracellular protein transport”, “vesicle-mediated transport”, and “protein transport”. Previous studies have demonstrated that the production of cryoprotectants such as glucose and urea was also increased as part of the response to dehydration stress in wood frogs^21^. Our finding suggests that proteins related to the transport biological process were upregulated during dehydration to aid cryoprotectant transport and distribution from liver to other organs in order to provide osmotic resistance against cell shrinkage below a critical minimum volume.

In order to have a better understanding of the metabolic changes supporting freeze tolerance, we focused on the significantly upregulated enzymes in treatment groups. Some of these enzymes were closely related to the freeze tolerance mechanism. For example, the sulfotransferases (SULT) are a group of transferase enzymes responsible for transferring sulfate groups from a common donor molecule 3ʹ-phosphoadenosine 5ʹ-phosphosulfate (PAPS) to an acceptor group of numerous substrates^41^. The anoxic condition promotes an increase in reactive oxygen species (ROS), leading to oxidative damage^42^. Dehydration can also cause oxidative stress either by elevating the production of ROS or by deactivating antioxidant enzymes^43^. Hossain et al*.* observed that the depletion of SULT results in the accumulation of ROS^44^. In our work, we found upregulation of the SULT enzyme in anoxic and dehydrated groups, suggesting that SULTs may be involved in the oxidative stress response.

Epoxide hydrolases (EH) catalyze the hydration of epoxides to trans-dihydrodiols and are very important enzymes in toxification–detoxification processes^45^. EH is a cytosolic enzyme that is highly expressed in liver^46^. It is known that glycogen synthase kinase-3beta (GSK3β) shows a strong increase in protein levels in liver of glucose-injected wood frogs^47^. Li et al*.* proved that soluble EH has a protective effect on oxidative damage by reducing ROS levels in rats and activating the PI3K/Akt/GSK3β signaling pathway^48,49^. In turn, activation of the GSK3β signaling pathway can lead to the overexpression of EH as a component of the stress response. Based on these potential roles of EH, it is not surprising that significant upregulation of EH in both dehydrated and frozen groups was identified in this study.

Other than SULT and EH, heme oxygenase (HO) was also identified as being involved in the cold stress response. Katori et al*.* showed that overexpression of HO-1 protected rat livers against cold ischemia–reperfusion injury (IRI)^50^. In addition, Venkatachalam et al*.* found that HO-1 can protect liver cells from cold ischemia in both in vitro and ex vivo models^51^. Ischemia occurs when blood supply falls below the body's normal demand, causing a shortage of oxygen. When the affected tissue is reperfused, a rapid and excessive accumulation of ROS occurs, potentially leading to IRI tissue damage^52^. Wood frogs remain frozen for weeks at a time and can also undergo multiple freeze–thaw cycles showing little or no tissue damage after thawing^16^. Our findings show HO upregulation in the frozen group, which suggests that HO potentially plays a key role in protecting tissues from damage under cold stress.

GO enrichment analysis of upregulated proteins in anoxic, dehydrated, and frozen groups using diverse stress and response terms revealed only three proteins: eosinophil peroxidase, epoxide hydrolase, and aconitate hydratase. The frog's liver appears to activate different adaptive molecular mechanisms under these three conditions but not many proteins are involved in the response to stress. In general, stress conditions can damage the structure and function of macromolecules and prolonged stress may cause death^53^. Therefore, *R. sylvatica* has developed a molecular adaptive mechanism to prevent potential damage to liver cells.

Antibodies specific to frog proteins are not commonly available commercially, so we chose an alternate method to validate the proteomic results. We chose enzymatic assays that are generally very sensitive and selective, for our validation method. Enzyme assays were used to assess the activities of three targets related to the freeze tolerance mechanism: glutathione S-transferases (GSTs), sorbitol dehydrogenase (SORD), and aldolase (ALDOA).

GSTs are a class of enzymes that catalyze the conjugation of glutathione, a major cellular antioxidant, to a wide variety of electrophilic compounds^54^. Under anoxic conditions, cells experience significant stress due to the lack of oxygen, which can lead to the accumulation of ROS and other toxic compounds^55^. Research has shown that GSTs are upregulated in response to cold stress in various organisms, including fungi^56^, plants^57,58^, and reptiles^7^. Interestingly, Willmore et al*.* showed that anoxia exposure caused significant reduction in the specific activity of liver GST in turtles^59,60^. Our findings from proteomic analysis showed significant upregulation of GSTs in anoxic, dehydrated, and frozen frogs. GST enzymatic activity was significantly higher in all three treatment groups as well. In the frozen condition, proteomics analysis revealed that GST was found to be 8.7-fold more expressed when compared to the control group. However, specific enzymatic activity increased only by about 1.8-fold. This discrepancy could be explained by the fact that enzymatic activity depends on the post-translational modifications as well as protein–protein interactions^61^. Notably, freeze-exposed wood frogs showed the highest protein expression and specific enzymatic activity of GST. Our results, along with previous studies, suggest that GSTs play a role in cellular detoxification by aiding the clearance of stress-induced ROS and consequently contributing to prevention of cellular damage.

Sorbitol dehydrogenase (SORD) is an enzyme involved in carbohydrate metabolism, transforming sorbitol (a sugar alcohol derived from glucose) into fructose, and providing an alternate source of energy^62^. Our proteomic results showed significant upregulation of SORD in anoxic and dehydrated groups, but not in the frozen group. However, SORD specific activity was significantly higher only in anoxic and frozen groups. This discrepancy between proteomics analysis and enzymatic activity assay could be due to the regulation of enzymatic activity by various post-translation modifications of SORD. The results of this study might suggest that this enzyme, as part of energy metabolism, can have a role in the adaptation of frogs to anoxic and dehydrated groups.

Aldolase (ALDOA) is a glycolytic enzyme that splits fructose 1,6-bisphosphate into two triose phosphate moieties that are further processed to pyruvate and then to either an anaerobic end point (e.g. lactate) or converted to acetyl-CoA that enters the Krebs cycle for aerobic catabolism. Both processes lead to ATP production but the aerobic route produces much more ATP^63^. Our findings from proteomic analysis showed that fructose-bisphosphate aldolase B was a unique protein in the anoxic group. It is reported that aldolase B is predominantly located in the liver and plays a role in the glycolysis pathway^64^. Our results using an enzymatic assay showed that ALDOA was detected in all groups, but its specific activity was significantly higher in anoxic and frozen groups. This result is also in agreement with previous studies^65,66^. Michaelidis et al*.* showed that aldolase activity in frogs initially increased within the first month of hibernation^65^, and Niu et al*.* revealed that the mRNA level of fructose-bisphosphate aldolase was significantly increased in hibernating frogs, *Nanorana parkeri*^66^. When considering the fact that regular glycolysis is inhibited in wood frogs during freezing^67^, these findings indicate that anaerobic glycolysis continues to function during hibernation. It appears that it is capable of supplying ATP for energy-requiring activities, but its efficiency is considerably lower compared to aerobic metabolic pathways. Therefore, significantly higher specific activity of aldolase in anoxic and frozen conditions could have roles in maintaining carbohydrate utilization during hibernation and retaining the functional ability to respond to any environmental changes.

Identified down-regulated proteins are related to different biological functions, but it was found that two proteins, malate dehydrogenase and oxoglutarate dehydrogenase, of the tricarboxylic acid cycle were affected for all three conditions. These results are in agreement with a previously reported investigation whereby pyruvate dehydrogenase from *Rana sylvatica* was found to be inhibited during 24 h freezing and 24 h anoxia. It has been suggested that the inhibition of this enzyme could reduce glycolytic flux and carbon entry into the tricarboxylic acid cycle as part of metabolic rate depression^68^.

Most enzyme-catalyzed reactions are temperature dependent, and most enzymes show reduced activity or are even inactivated at low temperatures^69,70^ The enzymatic assay results revealed that the frozen group displayed the relatively highest specific activity among the three enzymes tested: GST, ALDOA, and SORD. This increased enzymatic activity in the frozen group suggests the potential involvement of these enzymes in maintaining basic life functions of wood frogs under stress. In addition, these enzymes likely play a role in safeguarding tissues against damage under frozen conditions by maintaining the functionality of the most important metabolic pathways. The main limitation of this study is the lack of whole-genome sequencing of *Rana sylvatica*. The identification of proteins was aided by the genus rank taxonomic representation (*Lithobates*) in the Uniprot database instead of the species rank. This may cause potential misidentification of some proteins. Similar situations were applied to the gene ontology enrichment analysis, where the analysis was made using the database of a different species, American bullfrogs (*Rana catesbeianus*), that has the highest identified proteins under all conditions. Once the genome sequence of *Rana sylvatica* becomes available, it will be possible to obtain a more detailed image of proteome profiles using our reported data.

## Conclusion

The aim of this study was to analyze liver tissue from wood frogs under normal conditions and after anoxia, dehydration, or freezing exposures. The proteomic analysis confirmed differences in protein expression between controls and the three experimental groups. These results indicated that frogs developed many aspects of the molecular and cellular responses to these three stress conditions. Among differentially regulated proteins, three enzymes: GST, ALDOA, and SORD, were validated. The enzymatic assay results revealed that these enzymes showed a significantly higher specific enzymatic activity under anoxia and frozen treatments than the control group. Thus, our results strongly suggest that these enzymes might have important roles in maintaining normal metabolic function under extreme stress conditions. Overall, our data can provide a rich source of protein markers that may be used for further exploration of animal freeze tolerance.

### Supplementary Information

Supplementary Figures.

Supplementary Tables.

## Data Availability

All MS raw data were submitted to the PRIDE repository (Accession: PXD050540) at the European Bioinformatics Institute.
