# Supplementary material for: Proteomic analysis of Rana sylvatica reveals differentially expressed proteins in liver in response to anoxia, dehydration or freezing stress
Source: Sci Rep. 2024 Jul 4;14:15388. doi: 10.1038/s41598-024-65417-2 (PMC11224343; doi:10.1038/s41598-024-65417-2)
Supplement: Supplementary file 1 — Supplementary Figures. [file 41598_2024_65417_MOESM1_ESM.docx]

**Proteomic Analysis of *Rana sylvatica* Reveals Differentially Expressed Proteins in Liver in Response to Anoxia, Dehydration or Freezing Stress**

Yingxi Li ^1,2^, Zoran Minic ^2^, Nico Hüttmann ^2^, Abdullah Khraibah ^1^, Kenneth B. Storey ^3^, Maxim V. Berezovski ^1,2,^*

^1^ Department of Chemistry and Biomolecular Sciences, University of Ottawa, Ottawa, ON K1N 6N5, Canada

^2^ John L. Holmes Mass Spectrometry Facility, Faculty of Science, University of Ottawa, Ottawa, ON K1N 6N5, Canada

^3^ Department of Biology, Carleton University, 1125 Colonel By Drive, Ottawa, Ontario K1S 5B6, Canada

* Correspondence: maxim.berezovski@uottawa.ca (M.V.B.)

**Supplementary Figures**


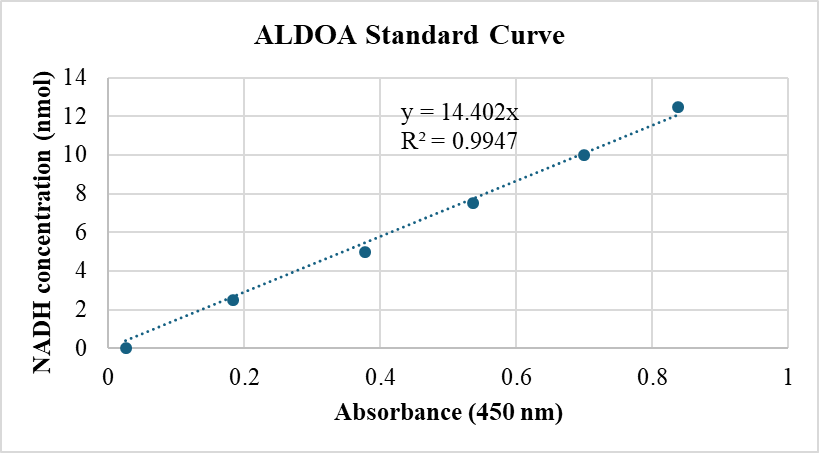

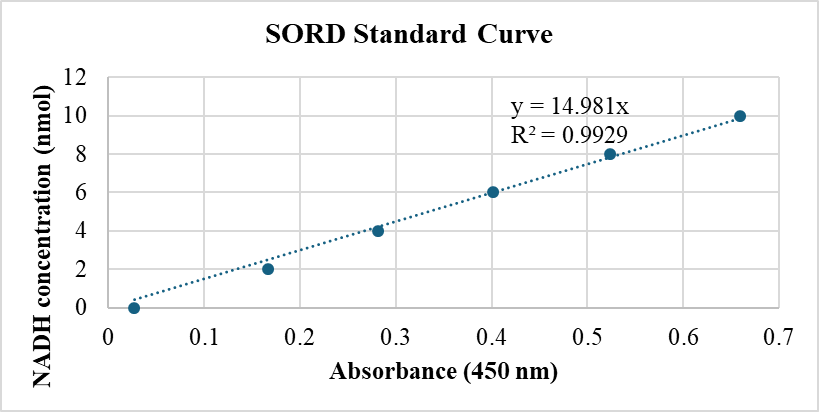


**A**

**B**

**Figure S1. The construction of standard curve for the detection of enzyme activity.** (A) Standard curve of aldolase. (B) Standard curve of sorbitol dehydrogenase.


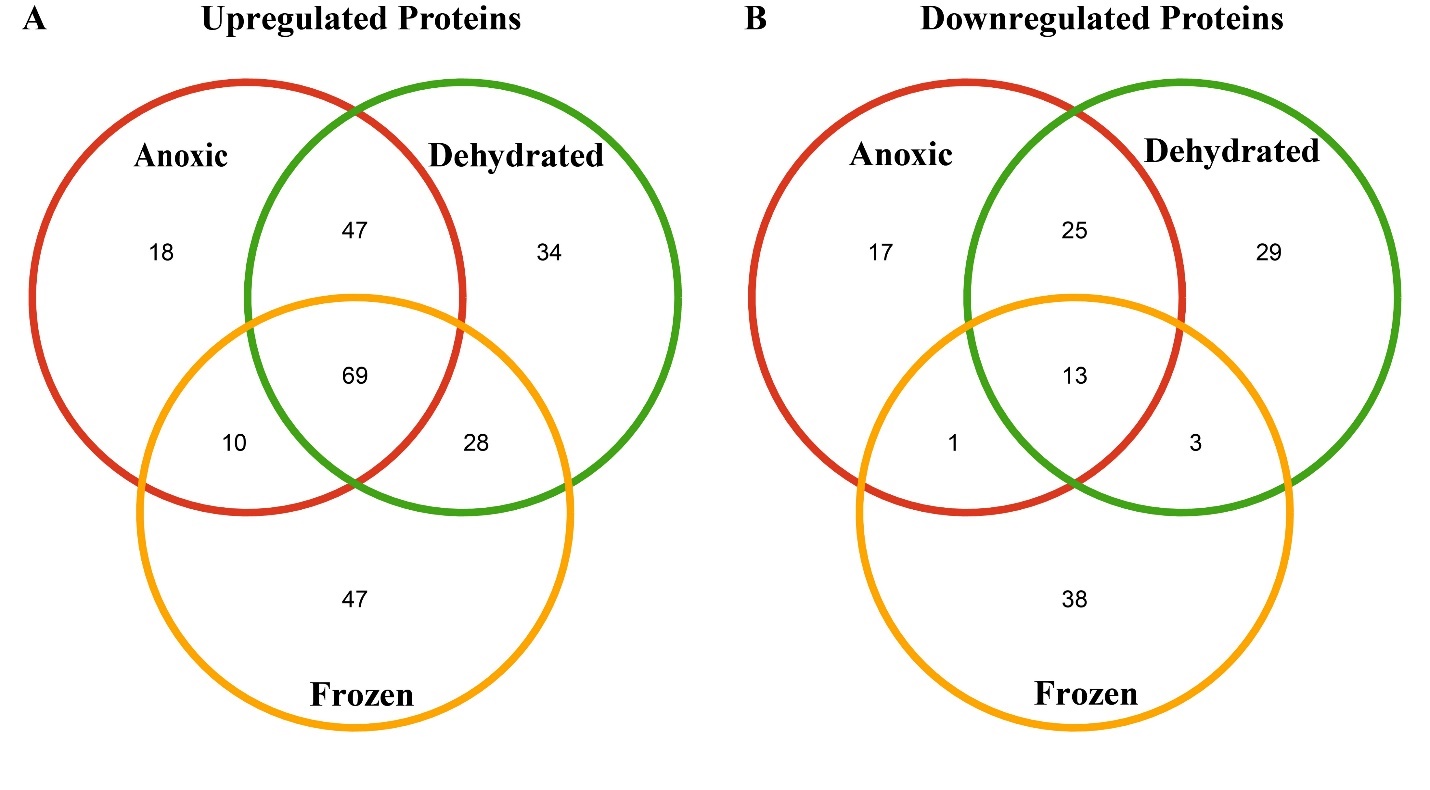
**Figure S2. Venn diagram of differentially expressed proteins from anoxic, dehydrated, and frozen groups.** (A) A Venn diagram of upregulated proteins. (B) A Venn diagram of the downregulated proteins.


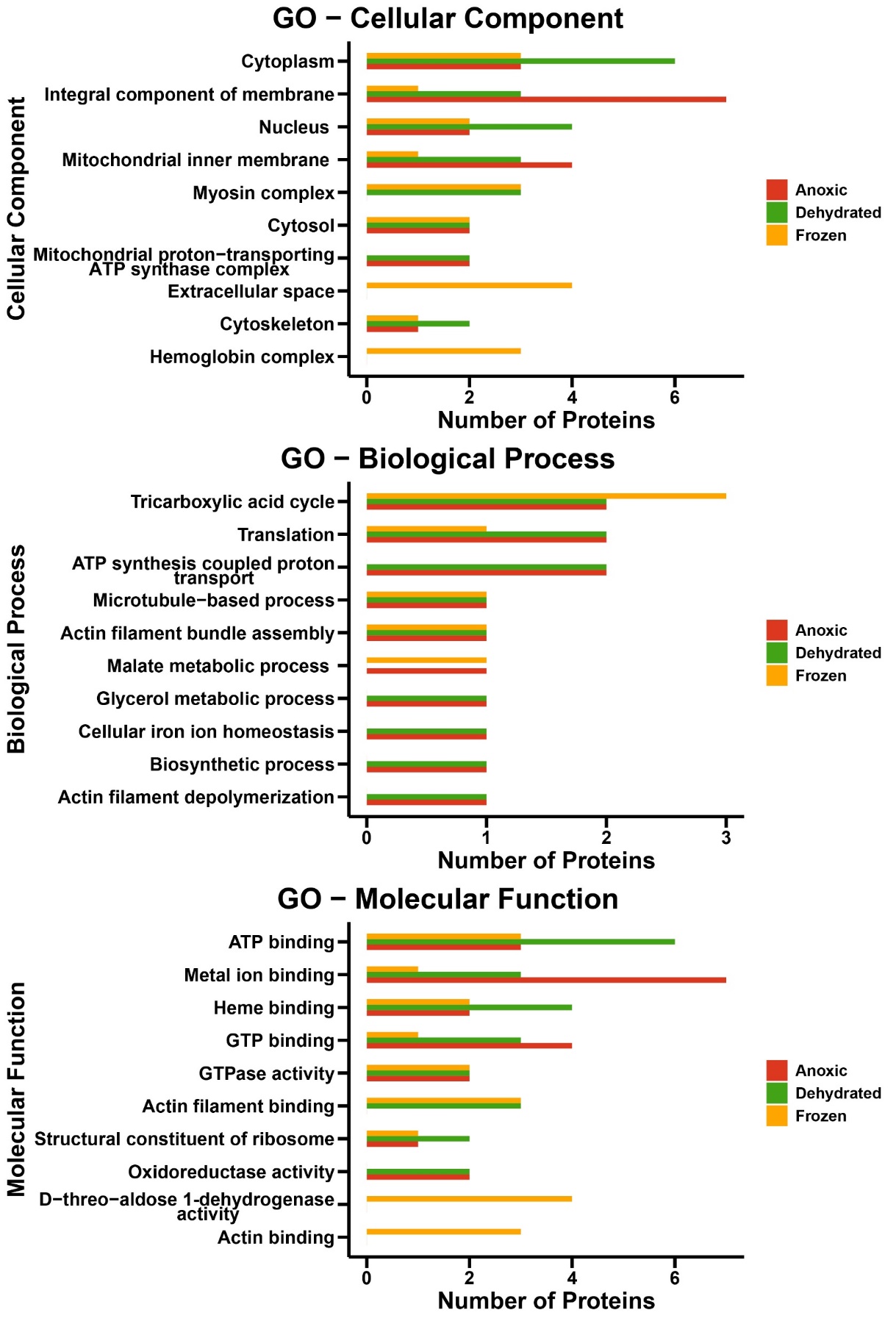


**Figure S3. Gene Ontology (GO) functional annotation labels for down-regulated proteins in anoxic, dehydrated, and frozen groups.** (A) Cellular Components, (B) Biological processes, and (C) Molecular functions.


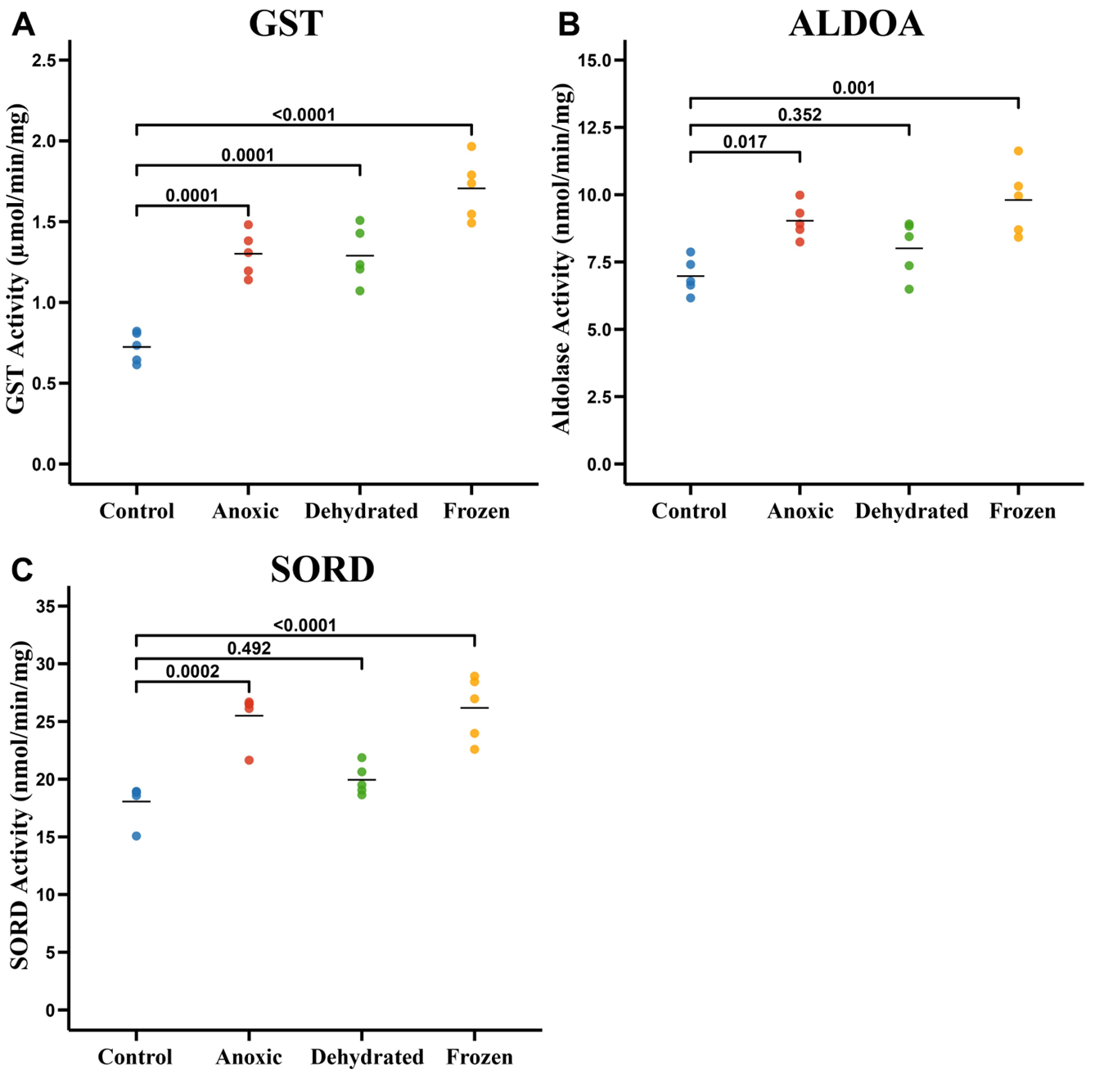


**Figure S4. Specific enzymatic activity** of (A) Glutathione S-transferase (GST), (B) Aldolase (ALDOA), and (C) Sorbitol dehydrogenase (SORD) from frog liver. This figure illustrates the comparative specific activities of multiple enzymes across various experimental treatments, statistically evaluated through one-way analysis of variance (ANOVA) coupled with Tukey's post-hoc test to identify significant differences at a threshold of p<0.05. The specific activity levels of all assessed enzymes under frozen and anoxic conditions were higher than those of the control group. An analogous significant increase in the specific activity of GST was observed in the dehydrated condition relative to the control. Conversely, the specific activities of ALDOA and SORD did not exhibit statistically significant differences between the dehydrated and control treatments.
